# Characterization of complete mitochondrial genome of *Pogonophryne albipinna* (Perciformes: Artedidraconidae)

**DOI:** 10.1080/23802359.2019.1698361

**Published:** 2019-12-12

**Authors:** Nazia Tabassum, Md. Jobaidul Alam, Jeong-Hoon Kim, Soo Rin Lee, Ji-Hyun Lee, Hyun Park, Hyun-Woo Kim

**Affiliations:** a Interdisciplinary Program of Biomedical, Mechanical and Electrical Engineering, Pukyong National University, Busan, Republic of Korea;; b Korea Polar Research Institute, Korea Ocean Research and Development Institute, Incheon, Republic of Korea;; c Department of Marine Biology, Pukyong National University, Busan, Republic of Korea;; d Division of Biotechnology, College of Life Sciences and Biotechnology, Korea University, Seoul, Republic of Korea

**Keywords:** Artedidraconidae, mitogenome, MiSeq, *Pogonophryne albipinna*

## Abstract

The complete mitochondrial genome of *Pogonophryne albipinna* was determined by the MiSeq platform, which was the first report in the family Artedidraconidae. The circular form of its mitochondrial genome was 17,086 bp, which contained the canonical eukaryotic 37 genes. The gene orders of *P. albipinna* was identical to the other icefish species, in which there was additional non-coding region and translocation of ND6 gene. Except for ATP6 gene and COI (GTG), 11 genes begin with the typical start codon, while incomplete stop codons (T– –) were identified in COII, ND4, and CytB. Phylogenetic tree with the currently known mitogenomes in suborder Notothenioidei showed that *P. albipinna* was located distinctly from those in Bathydraconidae and Nototheniidae forming a unique cluster as Artedidraconidae. The first complete mitochondrial genome of *P. albipinna* would be the fundamental data to understand the evolutional relationship of icefish species in the Antarctic Oceans.

Fish in the Artedidraconidae inhabit the deep sea of Southern Ocean, which comprise four genera including *Artedidraco*, *Dolloidraco*, *Histiodraco*, and *Pogonophryne*. Fish in the genus *Pogonophryne* is characterized by a weak development of bone ridges, plain snout rise, large number of rays in the pectoral and dorsal fins (Balushkin and Eakin 1998; Balushkin and Spodareva 2013, 2015). According to WoRMS (http://www.marinespecies.org/index.php), there are 25 species in the genus *Pogonophryne* but the complete mitochondrial genome sequence in the genus has not been reported yet. We here report the first complete mitochondrial genome from *Pogonophryne albipinna* by the combination of next-generation sequencing (NGS) technique and typical Sanger DNA sequencing of amplified PCR products.

*Pogonophryne albipinna* was collected from Antarctic Ocean (S74°37′27″, E164°14′19.5″) in 2018 as part of a research survey by National Institute of Fisheries Science (NIFS). Identification of the specimen was confirmed by both morphological characteristics as well as COI sequence identity (GenBank number: JN641104). The specimen and DNA of *P. albipinna* are stored at the Marine Biodiversity Institute of Korea (MABIK GR00002618). DNA extraction was carried out by DNA isolation kit (Abcam, UK) and further fragmented into size of 350 bp with the help of Covaris M220 Focused-Ultrasonicator (Covaris Inc., San Diego, CA). A library was constructed by TruSeq® RNA library preparation kit V2 (Illumina, San Diego, CA) and the nucleotide sequences were read by MiSeq platform (Illumina, San Diego, CA). Assembly of complete mitogenome was performed by Geneious^®^ 11.0.2 software (Kearse et al. 2012). Secondary structures of 22 tRNAs in the mitogenome were predicted by tRNAScan-SE software (Lowe and Chan 2016). Phylogenetic tree was constructed based on minimum evolution (ME) alogrithm using MEGA7.0 program (Kumar et al. 2016).

As a result of the assembly of MiSeq reads, the complete mitogenome of *P. albipinna* (GenBank Number: MN614417) was constructed and the control region with low complexity was reconfirmed by Sanger sequencing of a PCR product generated by sequence-specific primers. The complete mitogenome of *P. albipinna* (GenBank Number:MN614417) was 17,086 bp that encodes 37 genes including 13 protein, 22 transfer RNAs, 2 ribosomal RNAs. A higher percentage of A + T contents (51.71%) were observed compared with G + C contents (48.29%). Twenty-nine genes were situated on the heavy (H) strand while eight genes were on light (L) strand. Eleven genes were found with a typical start codon, while incomplete stop codons (T– –) were identified in COII, CytB and ND4. Among 22 tRNAs, tRNA^Ser-GCT^ did not display a typical clover-leaf structure. Traslocation of ND6 gene and the additional non-coding region were also identified as in the other antarctic notothenioids (Zhuang and Cheng 2010).

A phylogenetic tree was constructed to explore the evolutionary relationship of *P. albipinna* with the currently reported mitogenomes in suborder Nototheniodei. Phylogenetic tree showed that *P. albipinna* was distinctly located from those in Bathydraconidae and Nototheniidae forming a unique cluster as Artedidraconidae (Figure 1). This result would provide the fundamental data to understand the evolutional relationship of species in the suborder Nototheniodei.

**Figure 1.**
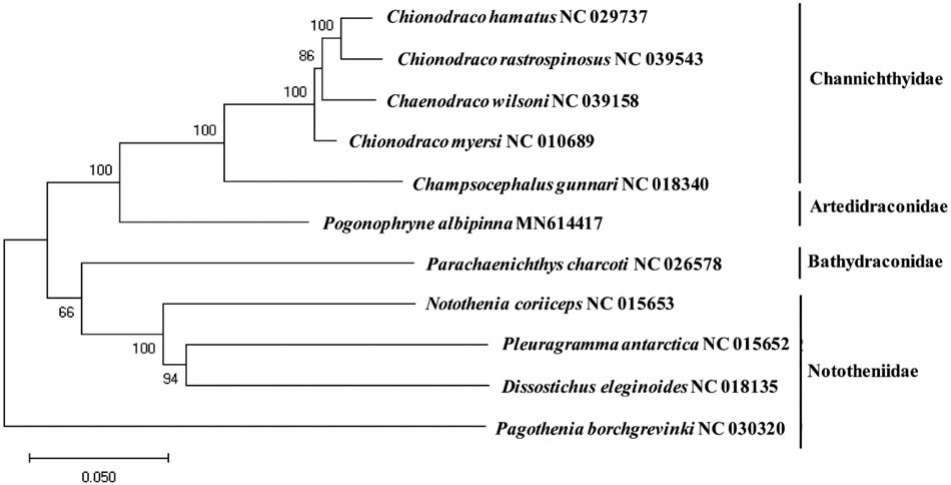
Phylogenetic relationship of *Pogonophryne albipinna* among the fish in suborder Notothenioidei. A phylogenetic tree was constructed with the currently reported complete mitochondrial genome in the order Perciformes by MEGA7 software using Minimum Evolution (ME) algorithm with 1000 bootstrap replications. GenBank accession numbers were shown followed by each species scientific name.
